# Enteric neural stem cell transplant restores gut motility in mice with Hirschsprung disease

**DOI:** 10.1172/jci.insight.179755

**Published:** 2024-07-23

**Authors:** Ahmed A. Rahman, Takahiro Ohkura, Sukhada Bhave, Weikang Pan, Kensuke Ohishi, Leah Ott, Christopher Han, Abigail Leavitt, Rhian Stavely, Alan J. Burns, Allan M. Goldstein, Ryo Hotta

**Affiliations:** 1 Department of Pediatric Surgery, Massachusetts General Hospital, Harvard Medical School, Boston, Massachusetts, USA.; 2 Department of Surgery, Boston Children’s Hospital, Boston, Massachusetts, USA.; 3 Drug Discovery Laboratory, Wakunaga Pharmaceutical Co. Ltd., Hiroshima, Japan.; 4 Stem Cells and Regenerative Medicine, UCL Great Ormond Street Institute of Child Health, London, United Kingdom.

**Keywords:** Cell biology, Gastroenterology, Neurodevelopment, Neuronal stem cells, Stem cell transplantation

## Abstract

The goal of this study was to determine if transplantation of enteric neural stem cells (ENSCs) can rescue the enteric nervous system, restore gut motility, reduce colonic inflammation, and improve survival in the Ednrb-KO mouse model of Hirschsprung disease (HSCR). ENSCs were isolated from mouse intestine, expanded to form neurospheres, and microinjected into the colons of recipient Ednrb-KO mice. Transplanted ENSCs were identified in recipient colons as cell clusters in “neo-ganglia.” Immunohistochemical evaluation demonstrated extensive cell migration away from the sites of cell delivery and across the muscle layers. Electrical field stimulation and optogenetics showed significantly enhanced contractile activity of aganglionic colonic smooth muscle following ENSC transplantation and confirmed functional neuromuscular integration of the transplanted ENSC-derived neurons. ENSC injection also partially restored the colonic migrating motor complex. Histological examination revealed a significant reduction in inflammation in ENSC-transplanted aganglionic recipient colon compared with that of sham-operated mice. Interestingly, mice that received cell transplant also had prolonged survival compared with controls. This study demonstrates that ENSC transplantation can improve outcomes in HSCR by restoring gut motility and reducing the severity of Hirschsprung-associated enterocolitis, the leading cause of death in human HSCR.

## Abstract 2

Enteric neural stem cell transplant increases peristaltic activity, reduces inflammation, and improves survival outcomes in mice with colonic aganglionosis.

## Graphical abstract

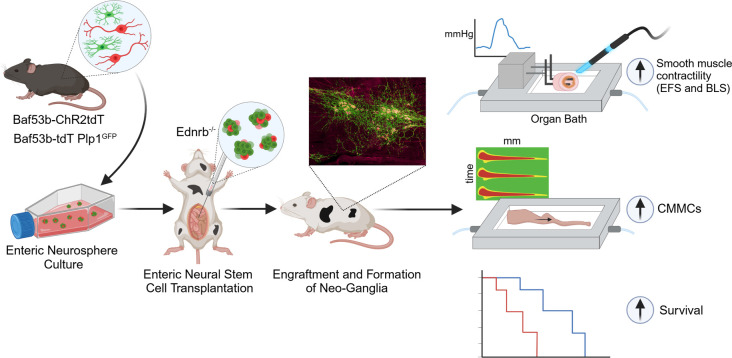

## Introduction

The enteric nervous system (ENS) refers to the complex interconnected network of neurons and glial cells that span the length of the gastrointestinal (GI) tract (1). Enteric neuropathies, in which enteric neurons are abnormal or absent, cause significant morbidity. Hirschsprung disease (HSCR) is a congenital enteric neuropathy affecting 1 in 5,000 live births and is characterized by the absence of ENS ganglion cells along variable lengths of distal bowel due to failure of neural crest–derived precursors to complete their colonization of the developing intestine (2, 3). The aganglionic bowel is functionally obstructed, and current treatment involves surgical resection of the aganglionic segment. While surgery is life-saving, many patients have persistent GI problems, including constipation, fecal incontinence, and enterocolitis (4, 5). Together with HSCR, other enteric neuropathies, including esophageal achalasia, gastroparesis, chronic intestinal pseudo-obstruction, and neurogenic constipation, cause serious morbidity, reduce quality of life, and result in significant healthcare costs (6). Despite the clinical importance of these conditions, current treatments are insufficient and largely aim to alleviate symptoms rather than to address the underlying pathophysiology. Cell-based therapy represents a novel approach that offers the potential to directly treat the cause of these neurointestinal diseases by replacing the absent or injured neurons (7–9).

Enteric neural progenitors have been isolated and cultured from laboratory animals (10–14) and humans (15), including patients with HSCR (16, 17), or generated from pluripotent stem cells (18). Following transplantation to animal models of enteric neuropathies, these cells have been shown to give rise to functioning neurons (19), improve gut motility (20–22), and improve outcomes in mice with HSCR (23, 24). While these seminal achievements demonstrate the potential of cell-based regenerative therapy for the treatment of enteric neuropathies, it remains unknown whether these cells can functionally integrate with the aganglionic smooth muscle to restore colonic peristaltic movement and reduce inflammation in HSCR. To address this question, we transplanted enteric neural stem cells (ENSCs) that express the light-sensitive ion channel channelrhodopsin-2 (ChR2) into the aganglionic segments of mice with HSCR. This optogenetic approach allowed us to selectively activate transplanted neural cells and evaluate the resulting gut contractile responses indicative of the establishment of successful neuromuscular connectivity. Further, using a glial/neuronal dual reporter mouse line, we investigated changes in the proportions of cell types present in neurospheres derived from the ENS before and after transplantation and demonstrated partial recovery of colonic peristalsis and reduction in inflammation in the aganglionic segment, leading to improved animal survival in mice with HSCR.

## Results

### ENSCs engraft, form neo-ganglia, and project neuronal fibers within the aganglionic colons of Ednrb-KO mice.

**Figure 1:**
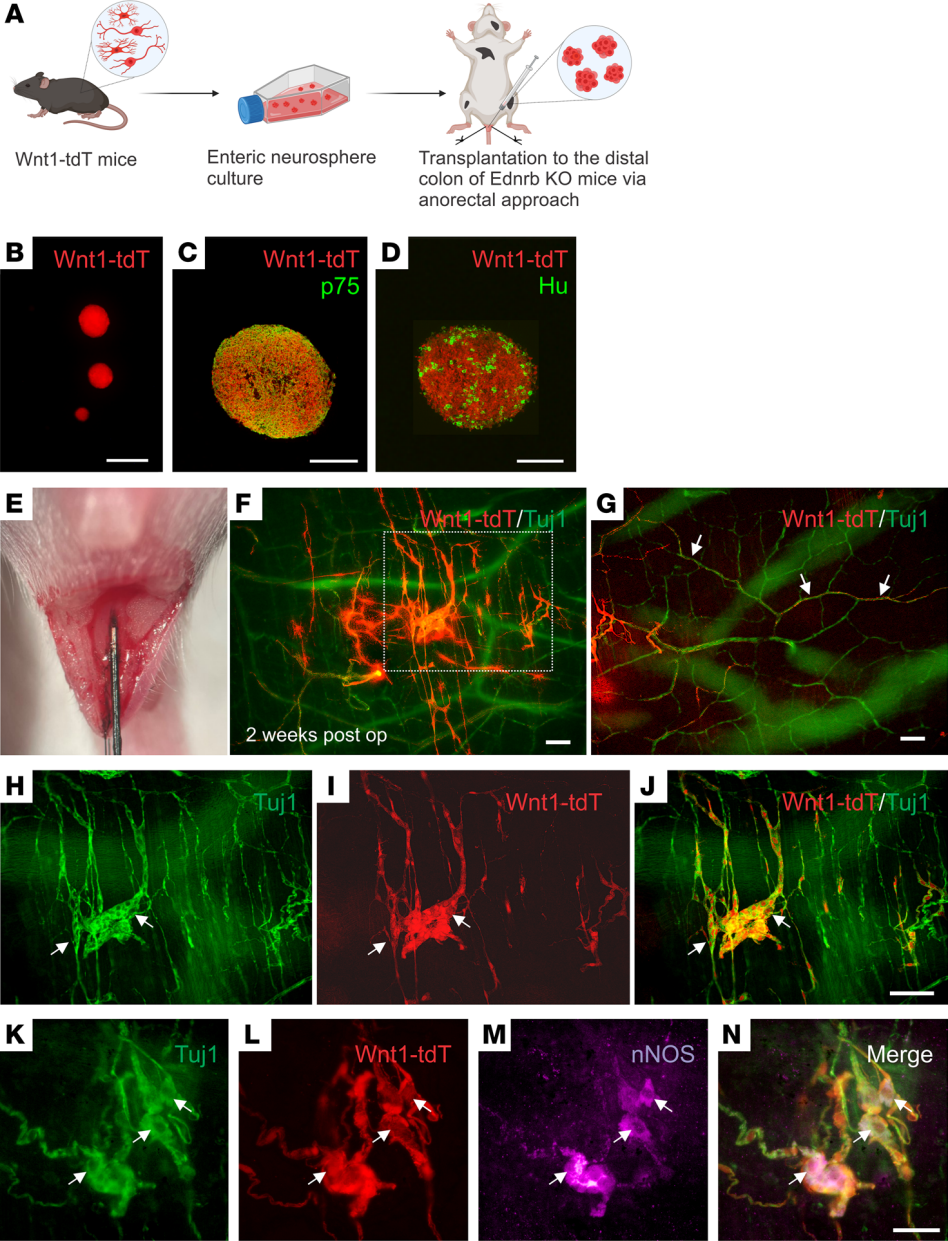
Transplant of Wnt1-tdT ENSCs to Ednrb-KO mice. Schematic of experimental overview (**A**), including isolation of ENSCs from the gastrointestinal tract of Wnt1-tdT mice, their expansion as neurospheres (**B**), and subsequent transplantation into the aganglionic distal colons of Ednrb-KO mice via anorectal needle injection (**A** and **E**). Enteric neurospheres contain p75^+^ neural crest cells (**C**) and Hu^+^ neurons (**D**). Transplanted cells were observed 2 weeks following surgery (**F**), projecting fibers along host-derived Tuj1^+^ extrinsic nerves (**G**, arrows) and forming neo-ganglia (**H**–**J**, arrows) that contain donor-derived nNOS immunoreactive neurons (**K**–**N**, arrows). Scale bars: 50 μm (**C**, **D**, and **K**–**N**), 100 μm (**F**–**J**), and 200 μm (**B**).

ENSCs were isolated from the GI tract of 2- to 3-week-old Wnt1-tdT mice, in which all neural crest–derived cells, including enteric neurons and glia, express tdTomato (tdT) (25) (Figure 1A). ENSCs were expanded in culture as enteric neurospheres (Figure 1, A and B) that contain p75^+^ enteric neural crest–derived cells (Figure 1C) and Hu-expressing neurons (Figure 1D). Neurospheres were transplanted via needle injection into the distal aganglionic colons of 7- to 10-day-old Ednrb-KO mice using an anorectal approach (Figure 1, A and E). Two weeks after surgery, transplanted tdT^+^ ENSCs were identified in recipient colons (Supplemental Figure 1A; supplemental material available online with this article; https://doi.org/10.1172/jci.insight.179755DS1) by costaining with the pan-neuronal marker, Tuj1 (Figure 1F, magnified view of the dotted box in F is shown in H; Supplemental Figure 1B). Transplanted cells formed clusters that resembled “neo-ganglia” and consisted of Tuj1^+^ neuronal cell bodies and fibers (Figure 1, H–J, arrows). ENSCs also projected neural fibers along the extrinsic-derived fibers that are a feature of the aganglionic colon (Figure 1G, arrows). Furthermore, neo-ganglia contained differentiated neurons that express nNOS (Figure 1, K–N, arrows), the enzyme required for producing nitric oxide (NO), which is involved in neurogenic relaxation of GI smooth muscle. These findings suggest that ENSCs can engraft; differentiate into enteric neurons, including enteric neuronal subtypes; and project fibers within the aganglionic gut environment in vivo, features that are necessary for normal ENS formation.

### ENSC transplantation restores motor responses in the aganglionic smooth muscle.

**Figure 2:**
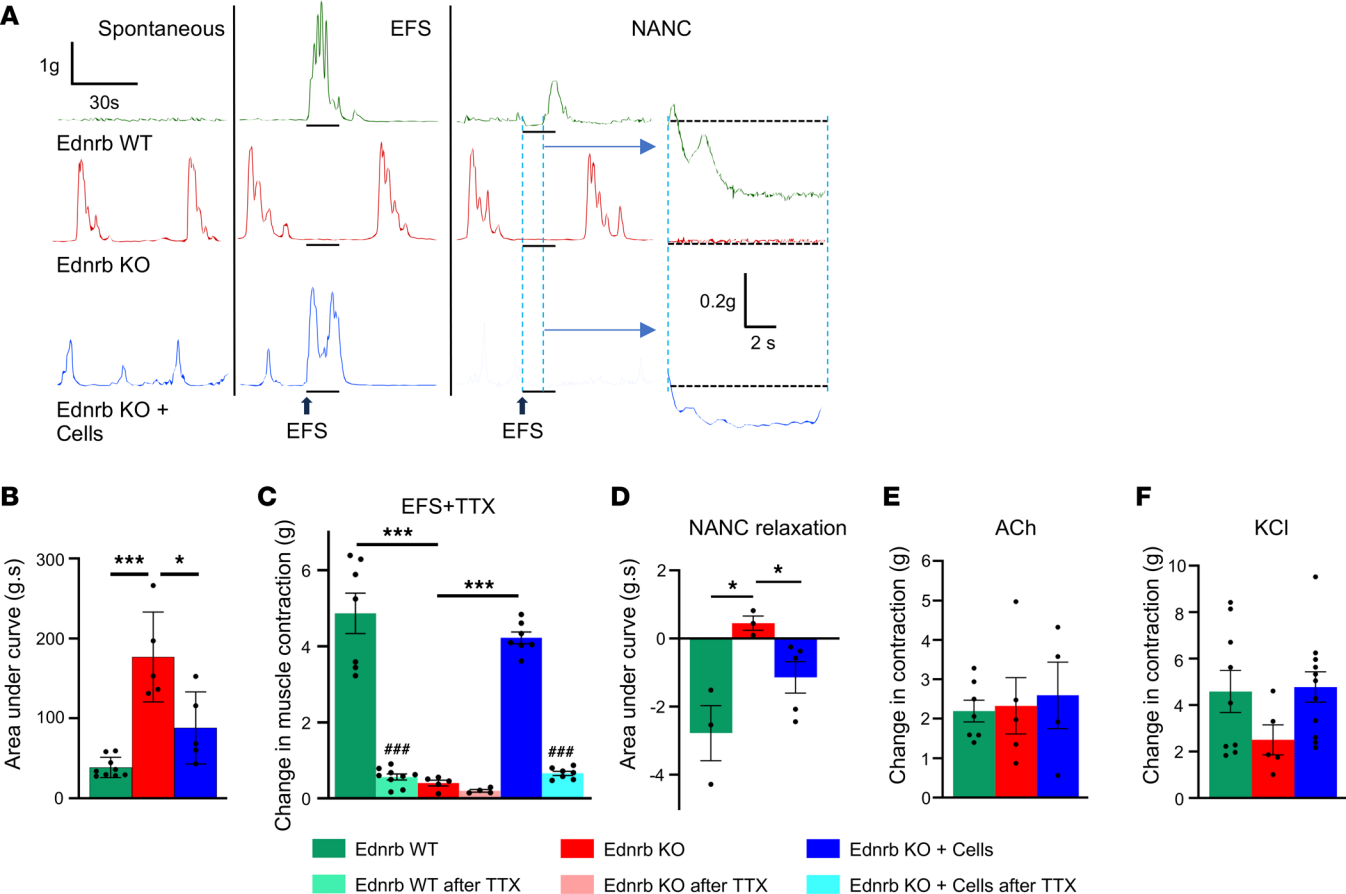
EFS demonstrates functional recovery of smooth muscle contractility in Ednrb-KO mice after cell transplantation. Representative traces of smooth muscle contractions during the spontaneous, after EFS, and under NANC conditions (**A**). Quantifications of spontaneous muscle contractility (**B**), EFS-induced contractility (**C**), and under NANC conditions (**D**) are shown. The amplitude of EFS contractions reflects maximal contractility as an absolute change from baseline and is markedly reduced in the presence of TTX (**C**). Effects of ACh (**E**) and KCl (**F**) on muscle activity. All the values represent the mean of 2–4 animals for each group, repeated 2–3 times. Data are shown as the mean ± SEM. Statistical significance was determined by the 1-way ANOVA with a post hoc Tukey’s test; *P < 0.05, ***P < 0.001, and ^###^*P* < 0.001 are statistically significant. EFS, electrical field stimulation; TTX, tetrodotoxin.

We next performed organ bath studies to determine the effect of ENSC transplantation on aganglionic smooth muscle activity. Aganglionic colonic smooth muscle excised from Ednrb-KO mice exhibited spontaneous, rhythmic, high-amplitude contractions at baseline (Figure 2A, red tracing). This activity was not observed in the ganglionated colon from normal, Ednrb WT mice (Figure 2A, green tracing). Importantly, the spontaneous contractions seen in Ednrb-KO colon were significantly reduced following cell transplantation (Figure 2A, blue tracing), as summarized graphically in Figure 2B. Following application of electrical field stimulation (EFS), an immediate contractile response was elicited in colonic smooth muscle from WT mice (Figure 2A, green tracing), whereas no response was observed in Ednrb-KO colon (Figure 2A, red tracing). However, after ENSC transplantation, the contractile response to EFS was restored in Ednrb-KO mice (Figure 2A, blue tracing). To test whether the contractile response was neurally mediated, the voltage-gated sodium channel blocker, tetrodotoxin (TTX) was added to the bath. In the presence of TTX, the contractile response was blocked in Ednrb WT as well as ENSC transplanted colon, confirming that the response was mediated by the transplanted enteric neurons. A quantitative analysis of these responses is provided in Figure 2C.

To determine if ENSC transplantation also restores the nitrergic inhibitory (relaxation) response in the aganglionic colon, EFS was performed in the presence of adrenergic and cholinergic antagonists, referred to as nonadrenergic, noncholinergic (NANC) conditions, to reveal the effects of NO, the main inhibitory neurotransmitter in the gut (26). In Ednrb WT colon, EFS elicited a relaxation response (Figure 2A, green tracing) but did not do so in Ednrb-KO colon (Figure 2A, red tracing). After cell transplantation, this inhibitory response was restored (Figure 2, A and D). Organ bath studies were also performed using aganglionic colon from sham-operated (vehicle injected) Ednrb-KO mice, and no responses were observed (Supplemental Figure 3). To determine the integrity and sensitivity of cholinergic receptors expressed by colonic smooth muscle preparations, 100 μM acetylcholine (ACh) was added to the organ bath and the force contraction was measured. Smooth muscles obtained from all 3 groups demonstrated an ability to contract in response to ACh, with no significant differences observed (Figure 2E). Addition of KCl also confirmed intact muscle contractile responses with no significant differences in responses among the 3 groups (Figure 2F). These findings indicate that transplanted ENSC-derived neurons restore functional contractile and relaxation responses in the recipient aganglionic colon.

### Optogenetics confirms functional neuromuscular connectivity between transplanted cell–derived neurons and aganglionic colonic smooth muscle.

**Figure 3:**
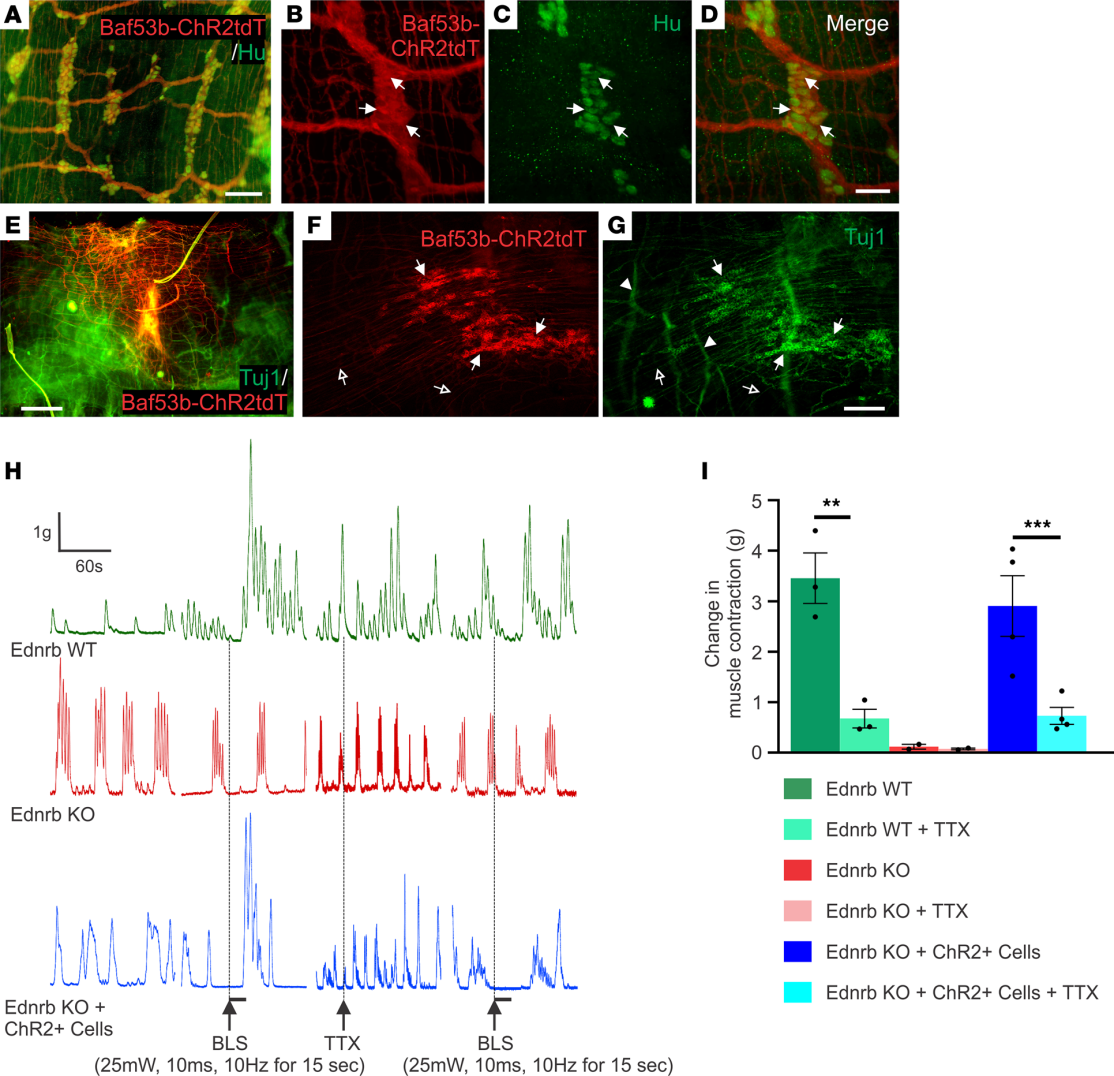
Optogenetics demonstrates neuromuscular connectivity between ENSCs and recipient aganglionic colon. Immunohistochemical evaluation of ENS in the Baf53b-ChR2tdT mice confirmed that Hu^+^ enteric neurons express ChR2tdT (**A**–**D**, arrows). Two weeks after surgery, transplanted cells were visualized (**E**). High-power images show that transplanted cells form neuronal cell clusters (**F** and **G**, arrows) with projecting fibers (**F** and **G**, open arrows), and hypertrophic nerve bundles (**F** and **G**, arrowheads) within the aganglionic colon. Traces depict spontaneous contractions and smooth muscle responses to BLS (**H**). While Ednrb-KO and WT colon show no response to BLS, transplantation of ChR2-expressing ENSCs leads to robust smooth muscle contraction (**I**), which is significantly reduced by the addition of TTX (**I**). Scale bars: 50 μm (**B**–**D**), 100 μm (**A**), 200 μm (**F** and **G**), and 500 μm (**E**). All the values represent the mean of 2–4 animals for each group, repeated 2–3 times. Data are shown as the mean ± SEM. Statistical significance was determined by the 1-way ANOVA with a post hoc Tukey’s test. **P < 0.01 and ***P < 0.001 are statistically significant. BLS, blue light stimulation; ChR2, channelrhodopsin-2; TTX, tetrodotoxin.

We next tested if neuromuscular responses could be demonstrated in transplanted gut by selectively activating only the transplant-derived neurons. ENSCs were isolated from *Baf53b:Cre;R26-Channelrhodopsin-2 tdTomato* (Baf53b-ChR2tdT) mice in which all neurons express the light-sensitive ion channel, channelrhodopsin-2 (ChR2), and thus can be activated by blue light stimulation (BLS). Immunohistochemical characterization of colonic muscle preparations dissected from 3-week-old Baf53b-ChR2tdT mice demonstrated complete overlap between Baf53b-tdT and Hu-immunoreactive enteric neurons (Figure 3, A–D), confirming that all gut-derived cells used for transplant are capable of responding to light stimulation. Following isolation and expansion of ENSCs from Baf53b-ChR2tdT gut tissues, we injected cells, as neurospheres, into the aganglionic colons of Ednrb-KO mice via the anorectal approach as described above. Ten days later, tdT^+^ ENSCs had formed neo-ganglia (Figure 3, E–G, arrows) containing Tuj1^+^ neurons with extensive neuronal processes (Figure 3, E–G, open arrows). Tuj1 staining also revealed hypertrophic nerve bundles within the aganglionic recipient colon (Figure 3G, arrowheads). Organ bath studies showed that aganglionic colon obtained from Ednrb-KO mice (Figure 3H, red tracing) or sham-operated Ednrb-KO mice (Supplemental Figure 3) exhibited spontaneous myogenic activity, as seen in Figure 2A but no contractile response to BLS. In contrast, BLS induced a robust contractile response following transplantation of ChR2^+^ neurons (Figure 3H, blue tracing). Quantitative comparisons demonstrated a significant increase in the amplitude of BLS-evoked muscle contractions in transplanted Ednrb-KO colons (Figure 3I). This effect was abolished by addition of TTX, confirming that the responses were mediated by transplant-derived neurons (Figure 3, H and I).

### Isolation, expansion, and characterization of ENSCs from Plp1GFP;Baf53b-tdT mice.

**Figure 4:**
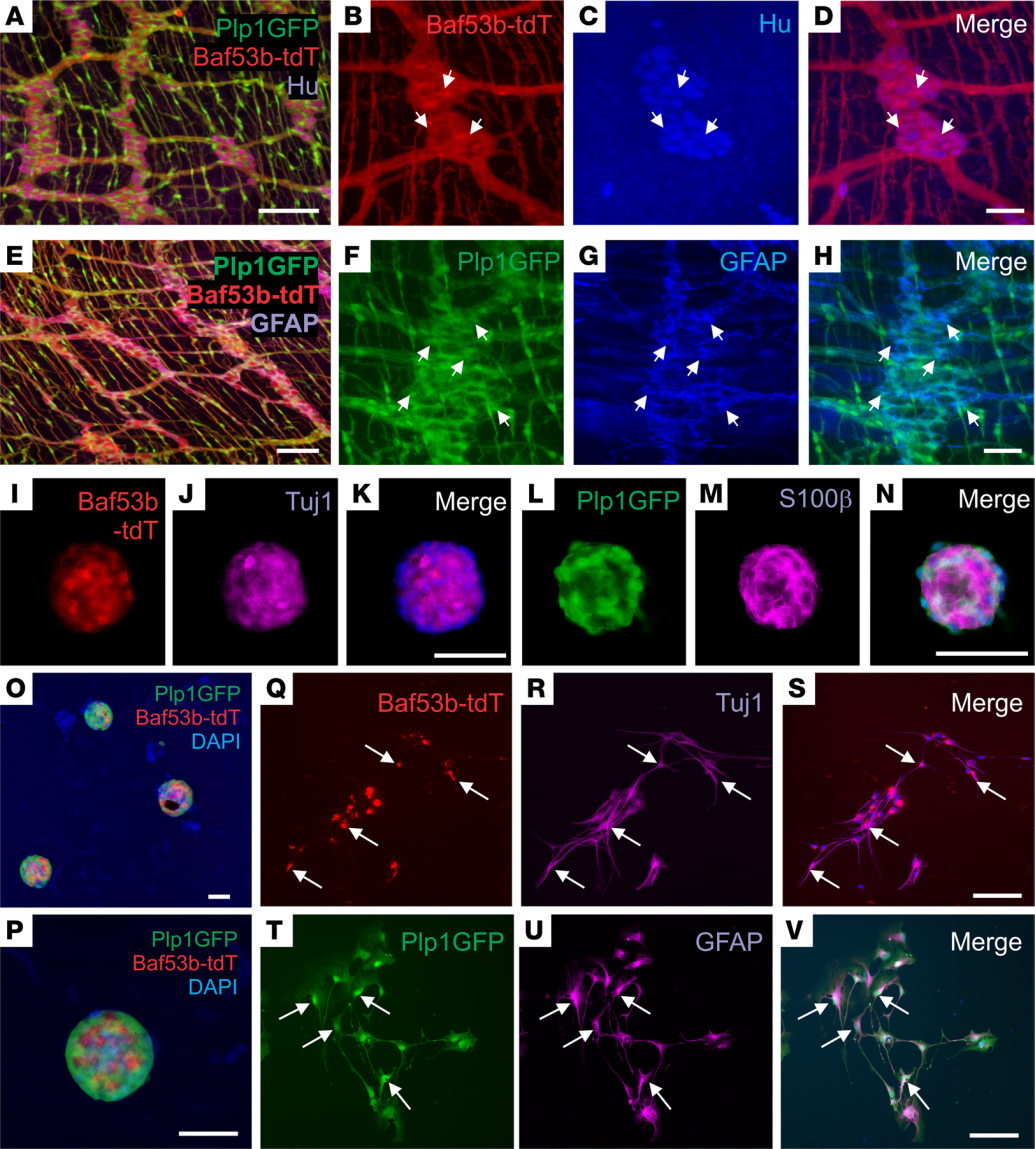
Isolation, expansion, and differentiation of ENSCs from Plp1GFP;Baf53b-tdT mice. Plp1GFP;Baf53b-tdT mice, in which Baf53b/Hu^+^ neurons express tdT (**A**–**D**, arrows) and PLP1/GFAP^+^ glial cells express GFP (**E**–**H**, arrows) were used to isolate ENSCs and generate enteric neurospheres (**I**–**P**), which express markers for neurons (Tuj1;J) and glia (S100β;M). Upon dissociation and culturing on fibronectin, neurospheres give rise to neurons (**Q**–**S**, Tuj1, arrows) and glial cells (**T**–**V**, GFAP, arrows). Scale bars: 50 μm (**B**–**D**, **I**–**K**, and **L**–**N**), and 100 μm (**A**, **E**–**H**, and **O**–**V**).

To allow more thorough cell characterization and determination of the fate of transplant-derived enteric neurons and glia, we generated a novel dual reporter transgenic mouse line (22) by crossing *Plp1GFP* (glial reporter) mice (27) with *Baf53b;R26-tdT* (neuronal reporter) mice (28). Immunohistochemical characterization of the longitudinal muscle-myenteric plexus (LMMP) obtained from 6-week-old Plp1GFP;Baf53b-tdT mice showed that Baf53b-tdT^+^ enteric neurons were immunoreactive for the pan-neuronal marker Hu (Figure 4, A–D, arrows), and Plp1GFP cells colocalized with GFAP-expressing enteric glial cells (Figure 4, E–H, arrows). ENSCs were isolated from these mice and expanded as neurospheres (Figure 4, I–P), which contained both Baf53b-tdT–expressing neurons and Plp1GFP-expressing glia. Immunostaining demonstrated colabeling of enteric neurons with Baf53b-tdT and Tuj1 (Figure 4, I–K) and of enteric glia with Plp1GFP and S100β (Figure 4, L–N). Dissociated neurospheres were plated on a fibronectin-coated surface, where they gave rise to neurons (Figure 4, Q–S, Tuj1, arrows) and glial cells (Figure 4, T–V, arrows), confirming the presence of neuroglial progenitors within the neurospheres.

### Neurospheres are enriched in enteric glia/progenitors.

**Figure 5:**
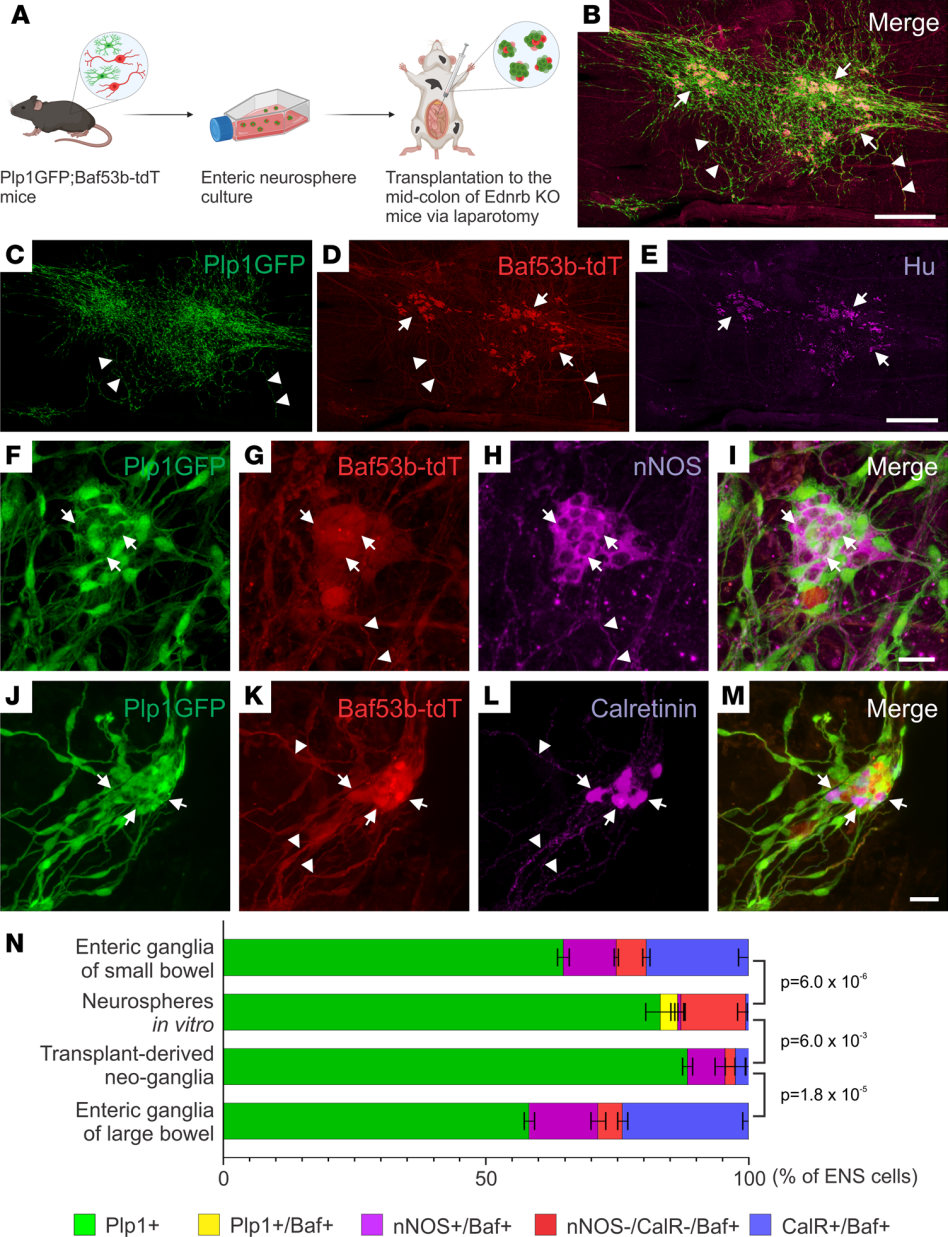
ENSCs transplanted into Ednrb-KO mice via laparotomy formed neo-ganglia that contain enteric neuron subtypes. The experimental design involves isolation of ENSCs from Plp1GFP;Baf53b-tdT mice, expansion as enteric neurospheres, and transplantation into the midcolon of recipient HSCR mice by multiple injections via laparotomy (**A**). Two weeks following surgery, transplanted cells are present in the aganglionic recipient colon (**B**). Many cell clusters contain neurons (**C**–**E**, arrows), and extensive fiber projections are seen (**C** and **D**, arrowheads). Transplanted ENSC-derived neo-ganglia contain nNOS-immunoreactive (**F**–**I**, arrows) and calretinin-immunoreactive (**J**–**M**, arrows) neurons with fibers (**G**, **H**, **K**, and **L**, arrowheads). Cell compositions in “Neurospheres in vitro” and “Transplant-derived neo-ganglia” were compared with those in the enteric ganglia of small or large bowel of 1- to 2-month-old WT mice (**N**). Statistical significance was determined by Fishers’ exact test. Scale bars: 25 μm (**F**–**M**) and 500 μm (**B**–**E**).

To determine ENSC fate, including generation of neural subtypes, prior to and following transplant to aganglionic colon in vivo, we isolated ENSCs from Plp1GFP;Baf53b-tdT mice and performed multiple injections of neurospheres into the midcolon of 2- to 3-week-old Ednrb-KO mice via laparotomy (Figure 5A). Recipient colons were examined 2 weeks following surgery. Transplanted ENSCs engrafted, migrated, and formed neo-ganglia (Figure 5, B–E, and Supplemental Figure 1, C and D) that contained Hu^+^ neuronal cell bodies with extensive fiber projections within the aganglionic gut environment. These observations are similar to our findings with Wnt1-tdT cell transplants (Figure 1) but with increased cell coverage due to performing multiple injections (4.9 ± 1.4 mm^2^ following single anorectal injection, *n* = 4, vs. 12.3 ± 4.5 mm^2^ after multiple injections via laparotomy, *n* = 3; Supplemental Figure 1E). We also observed that neural fibers projected extensively from the transplant sites and notably penetrated the muscle layers to reach the submucosal layer, similar to normal ENS (Supplemental Video 1). Further characterization of neo-ganglia formed by transplanted ENSCs demonstrated that both nNOS^+^ (Figure 5, F–I) and calretinin^+^ (Figure 5, J–M) enteric neuronal subtypes were present at 2 weeks after transplantation.

To determine the extent of neurogenesis and the fate of transplanted cells, quantitative evaluation of ENS composition of the neo-ganglia was performed and the results were compared with the cell types present within neurospheres in vitro prior to transplantation (Figure 5N and Supplemental Figure 2, A–A′′′) and to the endogenous enteric ganglia in 1- to 2-month-old WT mice (Figure 5N and Supplemental Figure 2, B and C). Cells within the neurospheres were predominantly (83.2%) Plp1^+^ glia/progenitors, while 13.6% were neurons, as shown by Baf53btdT expression. Interestingly, within the neurospheres before transplantation, a small population of cells (3.4%) was double-positive for Plp1GFP and Baf53btdT (Figure 5N and Supplemental Figure 2, A–A′′′, arrows), and only a small percentage of neurons expressed subtype markers (0.56% nNOS^+^ and 0.66% calretinin^+^, Figure 5N). After transplantation into aganglionic colon in vivo, the transplant-derived neo-ganglia were examined. The proportion of cells expressing Plp1GFP was essentially unchanged (88.4%, Figure 5N), and differentiated enteric neurons, expressing nNOS or calretinin, were present. However, the proportions of nNOS- and calretinin-expressing neurons within the neo-ganglia were significantly lower compared with those within normal enteric ganglia (7.1% nNOS and 3.0% calretinin in neo-ganglia vs. 13.1% NOS and 24.0% calretinin in “Enteric ganglia of large bowel,” Figure 5N). Since ENSCs were isolated from the small intestine, we also examined the ENS composition of enteric ganglia in the small bowel of 1- to 2-month-old mice and found that it contains 64.7% Plp1^+^ enteric glia, 10.1% nNOS^+^ neurons, and 19.5% calretinin^+^ neurons (Figure 5N and Supplemental Figure 2, D and E). This analysis suggests that neurosphere culture leads to an expansion of the Plp1^+^ glial population, which is known to include enteric neuronal progenitors, and a significant reduction in terminally differentiated enteric neuronal subtypes. These changes in cell proportions largely persist at 2 weeks following cell transplantation (Figure 5N), the latest time point we examined.

### Colonic dysmotility in the aganglionic colon is restored by ENSC transplantation.

We next evaluated whether ENSC transplantation restores colonic motility, as assessed by spatiotemporal mapping. This approach complements and extends the EFS and optogenetic experiments as, unlike those methods, it provides a quantitative whole-organ assessment of coordinated colonic contractility, which is a critically important endpoint for a regenerative cell therapy to treat HSCR. Two weeks following cell transplantation in vivo, the colon was removed, placed in an organ bath, and colonic migrating motor complexes (CMMCs) were evaluated.

**Figure 6:**
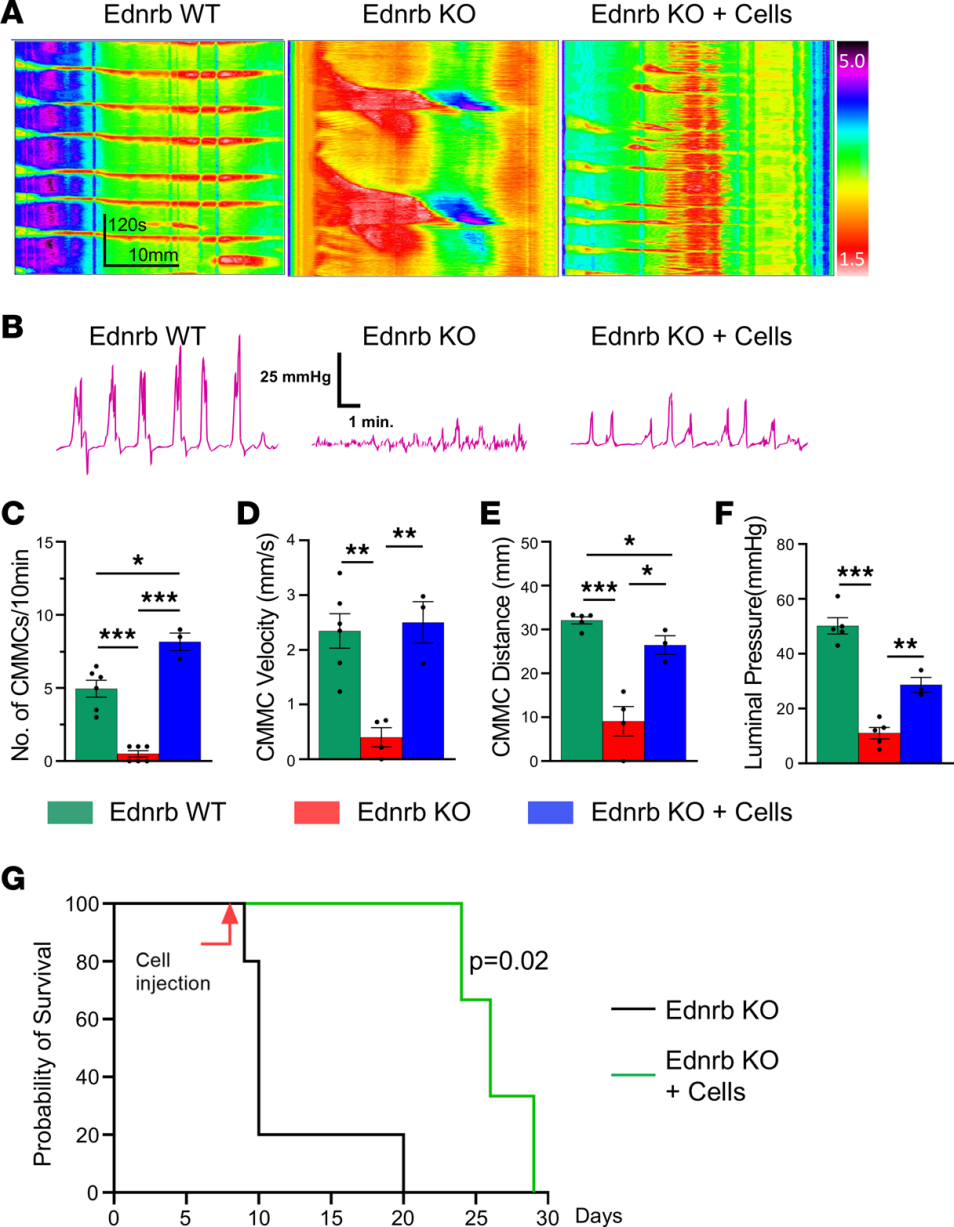
ENSC transplantation restores colonic motility in mice with HSCR and prolongs their survival. Representative spatiotemporal map kymographs generated from video recordings of colonic motility from Ednrb WT (*n* = 6), Ednrb-KO (*n* = 5), and Ednrb-KO + cells (*n* = 3) mice 2 weeks after cell transplant, depicting colonic contraction (red) and relaxation (yellow) along the length of the colon over time. The propagating CMMCs observed in WT mice are absent in KO mice but are partially restored following cell transplantation (Ednrb-KO + Cells) (**A**). Simultaneous intraluminal pressure recordings show effective colorectal contractility in WT mice, minimal pressure generation in Ednrb-KO mice, and significant restoration after cell transplant (**B** and **F**). CMMC frequency (**C**), velocity (**D**), and distance propagated (**E**) are all markedly increased in the Ednrb-KO + Cells group compared with the Ednrb-KO group. (**G**) Survival curve of Ednrb-KO mice that underwent ENSC transplantation (*n* = 3) or no treatment (*n* = 5). Statistical significance was determined by log-rank (Mantel-Cox) test (**G**). All the values represent the mean of 2–4 animals for each group, repeated 2–3 times. Data are shown as the mean ± SEM. Statistical significance was determined by the 1-way ANOVA with a post hoc Tukey’s test (**C–F**). *P < 0.05, **P < 0.01, and ***P < 0.001 are statistically significant.

Kymographs were generated from 10-minute video recordings of colons from Ednrb WT (Supplemental Video 2), Ednrb-KO (Supplemental Video 3), and Ednrb-KO + cells (Ednrb-KO mice with ENSCs transplanted into the mid-colon) (Supplemental Video 4) mice. Representative recordings are shown in Figure 6A. Analysis of the kymographs demonstrated that the number of CMMCs was significantly decreased in Ednrb-KO colons compared with Ednrb WT colons (5.0 ± 0.6 CMMCs in Ednrb WT vs. 0.5 ± 0.2 CMMCs in Ednrb-KO, *P* < 0.001, Figure 6C), and this was significantly improved following cell transplantation (8.2 ± 0.6 CMMCs in Ednrb-KO + cells, *P* < 0.001, Figure 6C). The significant reductions in CMMC velocity (0.4 ± 0.2 mm/s in Ednrb-KO vs. 2.3 ± 0.3 mm/s in Ednrb WT, *P* < 0.01, Figure 6D) and distance (9 ± 3.4 mm in Ednrb-KO colon, Figure 6E) were also improved by cell transplantation (Figure 6, D and E).

We also measured luminal pressure in the colon to determine whether the CMMCs were associated with a pressure change in the colonic lumen, a process necessary for the propagation of fecal contents. In Ednrb WT, a sharp increase in luminal pressure was observed during each CMMC, and this was absent in Ednrb-KO colon (Figure 6B). However, following ENSC transplant, luminal pressures were restored in the aganglionic colon, and these corresponded to the CMMCs that were observed in WT (50.2 ± 3.0 mmHg in Ednrb WT vs. 11.0 ± 2.0 mmHg in Ednrb-KO vs. 28.7 ± 2.7 mmHg in Ednrb-KO + Cells, Figure 6F). These findings indicate that ENSC transplantation significantly improves colonic motility in the aganglionic colon of mice with HSCR.

### ENSC transplant increases survival of Ednrb-KO mice via amelioration of enterocolitis.

Finally, to determine whether the restoration of dysmotility in Ednrb-KO mice by cell transplant has an effect on overall animal survival, we assessed survival time (days) following ENSC transplantation and compared these data with findings in naive Ednrb-KO mice. We found that ENSC transplantation significantly prolonged animal survival (median age; 10 days in naive, *n* = 5 vs. 26 days in ENSC transplant, *n* = 3, *P* = 0.02, Figure 6G). Because severe gut inflammation is a common and lethal complication of HSCR and is associated with early death of Ednrb-KO mice, we asked whether ENSC transplant could play a role not only in restoring gut motility, but in reducing colonic inflammation. Histological examinations of distal colon were performed to assess severity and depth of inflammation (Supplemental Figure 4A), and the degree of colonic inflammation was evaluated using enterocolitis scoring (29) (Supplemental Figure 4B). Consistent with previous reports (29), colonic inflammation was observed in Ednrb-KO mice (Supplemental Figure 4B), whereas ENSC transplantation markedly reduced the colonic inflammation score (Supplemental Figure 4B).

## Discussion

In this study, we utilized an animal model of HSCR to test the potential of ENSC transplant for the treatment of enteric neuropathies. We showed successful engraftment and formation of neo-ganglia within the aganglionic distal colons of Ednrb-KO mice following ENSC transplant. These cells gave rise to appropriate neuroglial phenotypes, including enteric neuron subtypes. Optogenetic-based electrophysiological analysis of recipient aganglionic colonic muscle demonstrated successful neuromuscular integration between ENSC-derived neurons and host smooth muscle. Cell transplant also resulted in partial restoration of CMMC activity of recipient colon, as demonstrated using spatiotemporal intestinal motility assays.

Over the past 2 decades, significant progress has been made in investigating the potential of cell replacement therapy for the treatment of enteric neuropathies, including HSCR (7, 30). Enteric neuronal progenitor cells have been isolated from the GI tract of laboratory animals (12, 19, 25, 31) as well as humans (17, 32) or derived from pluripotent stem cells established from mouse (33) or humans (24, 34), including patients with HSCR (18, 23). These cells have been transplanted into animal models, including models of enteric neuropathies, and successful cell engraftment, migration, and differentiation into functional neurons (14, 19) has been demonstrated, as has improved GI motility (20, 21, 24). These observations serve as proof of concept that cell therapy could be a promising therapeutic approach to replace missing or damaged enteric neurons and restore GI function. However, it is important to note that the precise mechanisms by which transplanted ENSCs elicit characteristics and properties typified by normal, functioning enteric neural cells are not fully understood. To date, we and others have shown successful integration of transplanted cells into the host enteric neuronal circuitry. Stamp et al. showed that optogenetic activation of transplanted cells evoked electric currents recorded from host colonic smooth muscle (14). ENSC transplantation also improved electromyographic activity (21) and inhibitory responses of recipient colonic smooth muscle (20, 21) in nNOS-deficient mice. However, most of these studies used ganglionated colon as recipient tissue; therefore, it remained unclear whether neurons derived from transplanted cells directly connected to recipient smooth muscle. In the current study, we transplanted ENSCs into aganglionic colon and observed contractile responses of recipient smooth muscle following optogenetic activation of transplanted ENSC-derived neurons. These findings clearly demonstrate a direct and functional connection between transplanted cells and recipient colonic smooth muscle. Optogenetics is a powerful tool for targeting specific cell types by light activation following delivery of the light-sensitive ion channel, channelrhodopsin, to cells of interest. We utilized the Cre-loxp transgenic strategy to selectively deliver ChR2 to enteric neurons and activate them following transplantation to aganglionic recipient colon in vivo. Our findings provide, for the first time to our knowledge, direct evidence that transplanted ENSC-derived neurons become integrated into the gut contractile circuitry by forming functional neuromuscular connections.

An interesting observation in our organ bath studies for identifying changes in gut contractility was that aganglionic smooth muscle displayed rhythmic myogenic contractions consistent with data previously reported by Barnes and Spencer (35). These authors used lethal-spotted (ls/ls) mice, another animal model of HSCR with hindgut aganglionosis, and performed organ bath mechanical recordings of colonic contractions. Myogenic motor patterns were identified in the aganglionic colon, and these phasic contractions were not blocked by the cholinergic transmission inhibitor hexamethonium, although the amplitude of contractions was significantly increased by nonselective NOS inhibitor, nitro-L-arginine. Post hoc immunohistochemical examination confirmed that the aganglionic segment of ls/ls mice does not contain neuronal cell bodies, suggesting that the cell bodies of these nerve fibers were likely located in the rostral ganglionated region (35). These findings indicate that some enteric cholinergic and nitrergic motor nerve fibers project into the aganglionic region and that increased muscle activity may be caused by inhibition of nitrergic inhibitory neural inputs. Interestingly, we observed that the myogenic phasic contractile activities were reduced following ENSC transplantation. Similar observations were previously made by Lindley et al. (36). These authors dissected and cultured aneural embryonic mouse hindgut with or without human-derived ENSCs for 1 week and observed a high frequency of spontaneous but uncoordinated contractions in the aneural embryonic hindgut. These myogenic activities were also significantly reduced by ENSC transplantation. Addition of TTX increased the rate of muscle contraction, suggesting that transplanted ENSC-derived neurons play an inhibitory role in suppressing this spontaneous myogenic contractile activity. In the current study, immunohistochemical examination showed the predominance of nNOS neurons within the neo-ganglia and EFS showed inhibitory nitrergic signaling in the aganglionic colon. This inhibition is important to achieve relaxation of the aganglionic segment in HSCR, because patients with HSCR are unable to pass stool due to an abnormally constricted distal aganglionic bowel.

In the current study, we observed substantially higher proportions of nitrergic neurons within the neo-ganglia that formed following ENSC transplantation. These cells appear to contribute to the inhibitory response in the recipient aganglionic smooth muscle because we observed a delay in contractile responses following optogenetic activation of transplanted cells (Figure 3C). It has previously been shown that optogenetic stimulation of excitatory cholinergic enteric neurons elicits immediate contractile activity of the colonic smooth muscle (37, 38). In our model, the delayed contraction could be due to a rebound response following the release of inhibition caused by activation of the predominant nNOS neurons in the neo-ganglia. Stamp et al. (14) reported that approximately 40% of Hu^+^ neurons within neurospheres prior to transplant were immunoreactive for nNOS. While limited data are available to determine the cell fate of neurospheres and ENSCs following transplantation into mouse colon in vivo, preferential differentiation of ENSCs into nitrergic neurons may be occurring in our model. Bergner et al. (39), looking at the emergence of various enteric neurochemical subtypes during development, found that a small fraction of developing myenteric neurons express nNOS transiently. A more recent study using transcriptomic profiling strategies showed that a switch from nitrergic to cholinergic enteric neurons occurs postnatally (40). Since nitrergic neurons have been shown to appear earlier in ENS development in myenteric ganglia than cholinergic neurons (41–43), the predominance in nNOS neurons observed in our current study may be temporal as our immunohistochemical analysis was performed 7–10 days following transplantation, and this pattern may change as the transplanted cells further mature over time as observed. Although additional studies are needed to determine such cell fate development and change longitudinally, the Ednrb-KO mouse model of HSCR used in this study is lethal in the early postnatal stages, which limits the ability to perform longer term follow-up investigations beyond 2–3 weeks.

Another important outcome demonstrated in the current study was the partial restoration of CMMCs in the aganglionic colon following cell transplantation. As previously reported, optimization of the cell delivery method by multiple injections into the colonic wall has been shown to be beneficial for maximizing cell coverage and has led to improved colorectal motility (21). In the current study, multiple injections of ENSCs maximized in the cell coverage up to 16.8 mm^2^ at 2 weeks after transplantation (Supplemental Figure 1E); however, a large area of the middistal colon remained aganglionic, as shown in Supplemental Figure 1, B and C. Nonetheless, ENSC transplantation resulted in a partial recovery of CMC activity, likely due to extensive but fine neural fiber projections extending beyond the cell coverage that were not easily detected. Our previous work (19) showed that the area covered by graft-derived fibers was 2 times larger than that by cells (20 mm^2^ by fibers vs. 10 mm^2^ by cells, 16 weeks after surgery). McCann et al. transplanted ENSCs into the distal colons of nNOS^–/–^ mice in vivo and observed that neural fibers extended from transplanted ENSCs up to 42.4 mm whereas ENSCs migrated and colonized around 6 mm^2^ of the colonic surface area 4 weeks following transplantation (20). These graft-derived fibers may connect with host ENS, which can act as the intrinsic neural circuit, a key element for the generation of CMC activity along the uncolonized colonic segment (44). On the other hand, recent studies using PSC-derived enteric neural progenitor cells showed that a single injection into the cecal wall was sufficient to improve gut function as well as survival in HSCR mice (23, 24). These studies also reported the striking migratory ability of transplanted enteric neural progenitors; therefore, optimal delivery methods may depend on the type of cell used for transplant, with more injections required for one and not the other. It is well documented that peristalsis of the aganglionic distal colon is absent or ineffective in mice and humans with HSCR (35, 45). Although we have confirmed, using EFS and optogenetics, that transplanted ENSC-derived neurons successfully integrated and formed functional neuromuscular connections with aganglionic smooth muscles and partially propagated CMMCs, cell transplants are yet to demonstrate restoration of fully propagated peristaltic waves through the length of the previously aganglionic colon.

Interestingly, along with this partial restoration of function, we saw increased survival of Ednrb-KO mice following cell transplant and improved enterocolitis scores. Patients with HSCR are at risk for developing the devastating bowel inflammatory disorder, Hirschsprung associated enterocolitis (HAEC), which represents the leading cause of serious morbidity and mortality in those children (46). Infants who develop HAEC are at risk of severe, long-term bowel dysfunction (47) and have a mortality rate of 0.3%–2.3% (48, 49). As no single etiology has been identified, the clinical entity of HAEC likely results from several alterations in intestinal homeostasis, including, (a) dysfunction of intestinal barrier, (b) abnormal innate immune responses, and/or (c) altered microbiome in patients with HSCR (50, 51). Treatment tends to remain empiric and directed toward alleviating symptoms rather than addressing the pathophysiology (46). In the current study, ENSC transplantation appears to alleviate colonic inflammation, suggesting the potential of an antiinflammatory role of the ENS. Extensive evidence has shown that parasympathetic innervation elicits antiinflammatory effects by an interplay between ACh-releasing memory T cells and α7-nicotinic ACh receptor^+^ (α7nAChR^+^) splenic macrophages (52–54). More recently, we and others have shown that cholinergic myenteric neurons and α7nAChR^+^ muscularis macrophages are involved in the intestinal cholinergic antiinflammatory pathway (CAI) (55, 56). A recent human cohort study also reports a correlation in the lack of cholinergic mucosal innervation and higher incidence of HAEC (57); therefore, restoration of the cholinergic system by transplantation of ENSCs to the aganglionic colon may reduce colonic inflammation via activation of resident macrophages. However, further dissecting and understanding how cell transplant affects the motility mechanisms that underly normal gut function, as well as its effect on inflammation in the aganglionic colon, will be required to maximize the clinical benefit of cell therapy for HSCR.

## Methods

### Sex as a biological variable.

Our study examined male and female animals, and similar findings are reported for both sexes.

### Animals.

The various breeding schemes and genotypes of controls are summarized in Table 1. *Wnt1:Cre* mice (stock no. 003829 and stock no. 009107), *R26-tdT* reporter mice (stock no. 007914), and *R26-ChR2tdT* reporter mice (stock no. 012567) were purchased from The Jackson Laboratory. *Wnt1:Cre* mice were crossed with *R26-tdT* and *R26-ChR2tdT* reporter mice to generate *Wnt1:Cre;R26-tdT* (annotated as Wnt1-tdT) and *Wnt1:Cre;R26-ChR2tdT* (annotated as Wnt1-ChR2) mice, respectively.

We also generated Plp1GFP;Baf3b-tdT mice (22) in which enteric glial cells express GFP and neural crest–derived enteric neurons express tdTomato by crossing *Plp1GFP;Baf53b:Cre* mice with *R26-tdT* mice. Plp1GFP mice (58) were gifted by Wendy Macklin, University of Colorado, Denver, Colorado, USA.

### Isolation and expansion of ENSCs.

ENSCs were isolated from Wnt1-tdT, Baf53b-ChR2tdT, or Plp1GFP;Baf53b-tdT mice as previously reported (25, 59). Briefly, LMMP was separated from small intestine of 2- to 3-week-old mice. Enzymatic dissociation was achieved using dispase (250 μg/mL; StemCell Technologies) and collagenase XI (1 mg/mL; Sigma-Aldrich) at 37°C for 40 minutes. Single cells were isolated by filtration through a 40 μm filter and plated at 100,000 cells/mL in a 25 cm^2^ flask in mouse proliferation media, consisting of DMEM (Gibco) supplemented with 10 ng/mL Insulin-like growth factor-1 (StemCell Technologies), 10 ng/mL basic fibroblast growth factor (StemCell Technologies), 1% penicillin/streptomycin (Gibco), 1% N2 supplement (Gibco), 2% B27 supplement (Gibco), 50 μM 2-Mercaptoethanol (Gibco), and 75 ng/mL Retinoic acid (Sigma-Aldrich). After 7 days, primary neurospheres were obtained and used for transplantation experiments.

### Transplantation of ENSCs to the mouse gut in vivo.

Seven- to 10-day-old Ednrb-KO and Ednrb WT mice were used for in vivo transplantation experiments. Recipient mice were anesthetized by isoflurane inhalation. A perianal or midline abdominal skin incision was made. Cell suspension was prepared at 10 neurospheres per μL (for multiple injections via laparotomy) or 30 neurospheres per μL (for single anorectal approach), and 3 μL was injected per site. After cell injection, sites were tattooed with India ink for later identification. Sham operation consisted of injection of 3 μL vehicle only through the same approach as above.

### Immunohistochemistry.

Immunohistochemistry was performed on recipient mouse colon, as previously described (22, 25). Whole-mount preparations of the LMMP and enteric neurospheres were fixed in 4% paraformaldehyde. Whole-mount LMMP or neurosphere preparations were permeabilized with 0.1% Triton X-100 and blocked with 10% donkey serum. Primary antibodies were diluted in 10% donkey serum and included goat anti-GFAP (1:200, Abcam, ab53554), human anti-HuC/D (Anna1, 1:16000, gifted by Vanda Lennon lab (Mayo Clinic, Rochester, Minnesota, USA), mouse anti-HuC/D (1:50, Invitrogen, A-21271), rabbit anti-rabbit anti-calretinin (1:200, Invitrogen), rabbit anti-neuronal NO synthase (nNOS; 1:200, Thermo Fisher), rabbit anti-p75 neurotrophin receptor (P75; 1:400; Promega), rabbit anti-S100 β antibody (1:100, Abcam, US), and mouse anti-neuronal class III conjugated β-tubulin (Tuj1; 1:400; Covance). Secondary antibodies included anti-rabbit IgG (1:500; Alexa Fluor 488; Fisher Scientific Life Technologies) and anti-human IgG (1:200, Alexa Fluor 594; Fisher Scientific Life Technologies). Cell nuclei were stained with DAPI (Vector Labs) and mounted with aqua-poly/mount (Fisher Scientific Polysciences Inc.). Images were taken using Nikon A1R laser scanning confocal microscope (Nikon Instruments), Nikon AXR confocal microscope (Nikon Instruments), or Keyence BZX-700 All-In-One Microscopy system (Keyence America).

For H&E staining, paraffin-embedded colonic samples were sectioned at 5 μm, deparaffinized, cleared, and rehydrated in graded solutions. Sections were immersed in xylene (3 × 4 minutes), 100% ethanol (3 minutes), 90% ethanol (2 minutes), and 70% ethanol (2 minutes) and rinsed in tap water. They were then immersed in hematoxylin (4 minutes), rinsed in Scott’s tap water (1 minute) and eosin (3 minutes), and rinsed again in tap water. This was followed by incubation in 100% ethanol (2 × 1 minute), xylene (2 × 3 minutes), and mounting on glass slides with distyrene plasticizer xylene mountant.

### Measurement of smooth muscle activity using EFS.

Experiments were performed using standard organ bath techniques as described previously (22, 60). Briefly, freshly excised distal colon was quickly placed in Krebs solution. Tissue was cut into 5 mm rings and mounted between 2 small metal hooks attached to force displacement transducers in a muscle strip myograph bath (model 820 MS; Danish Myo Technology) containing oxygenated Krebs solution at 37°C. The rings were gently stretched to deliver a basal tension of 0.5 g and were equilibrated for 30–45 minutes, with the Krebs changing every 20 minutes. For EFS, colon segments were then stimulated with pulse trains of 40–50 V for 15 seconds, with pulse duration of 300 μs, at a frequency of 5 Hz using a CS4+ constant voltage stimulator with Myo Pulse software (Danish Myo Technology) in the presence or absence of NANC conditions (1 μM atropine, 1 μM phentolamine hydrochloride, and 1 μM propranolol hydrochloride). Force contraction of the circular smooth muscle was recorded and analyzed using a Power Lab 16/35 data acquisition system (ADInstruments) and Lab Chart Pro Software v8.1.16 (ADInstruments). ACh (100 μM, Sigma-Aldrich) was added to the organ bath to measure maximum contraction. Muscle contraction was also recorded in the presence of TTX (0.5 μM, Alomone Labs), a voltage-gated sodium channel blocker. Tissue viability and integrity were confirmed at the end of the experiment by measuring contraction response to 60 mM KCl.

### Optogenetics.

Segments of the transplanted colon were dissected and prepared as above. BLS was applied from a diode-pumped solid-state laser system (470 nm, 200 mW, model number MDL-III-470; OptoEngine, LLC). Trains of light pulses (20 ms pulse width, 20 mW/mm^2^ light intensity, 10 Hz, 15-second train duration) were focally shone on the serosal surface of the transplanted colon in the organ bath via a glass fiberoptic (200 μm diameter). Light intensity was assessed using Power and Energy Meter Interface (PM100USB, Thorlabs) and Standard Photodiode Power Sensor (S121C, Thorlabs).

### Data acquisition and analysis of organ bath studies.

Baseline maximum value was taken from 60 seconds of data 1 minute prior to EFS or BLS, and maximum changes for contraction were taken from 60 seconds of data starting at stimulus and expressed as absolute changes from baseline maximum values. EFS and BLS were repeated 3 times in 5-minute intervals, and maximum response was calculated as a mean of 3 responses. The AUC was analyzed from 60 seconds of data during baseline recording. The baseline maximum value was determined from 60 seconds of data collected 1 minute before EFS or BLS. The maximum changes due to contraction were measured from 60 seconds of data starting at the beginning of the stimulus. These changes were then expressed as absolute differences from the baseline maximum values. The AUC, minus baseline, during first 10 seconds of the EFS period was determined as NANC relaxation (21).

### Measurement of colonic luminal pressure.

The entire colon was removed from Ednrb-KO + cells mice 10 days after cell transplantation and age-matched Ednrb WT and Ednrb-KO mice and placed in an illuminated organ bath and left to naturally expel fecal content. The empty colon was cannulated at both ends and arranged horizontally in an organ bath chamber. The proximal end of the colon was connected to a reservoir of Krebs solution (maintained at 15 mL), the height of which was adjusted to change the intraluminal pressure (0–10 mmHg). The distal end was attached to a polyethylene tubing connected directly to pressure transducer (CWE Inc.). All signals were digitized and recorded using Power Lab 16/35 data acquisition system (ADInstruments) via Lab Chart Pro Software v8.1.16 (ADInstruments).

### CMMC video recording and generation and analysis of spatiotemporal maps.

Organ baths were continuously superfused with Krebs solution at 36.5 ± 0.5°C and bubbled with carbogen gas (95% O_2_/5% CO_2_), and preparations were left to equilibrate for 15 minutes. Following a 15-minute equilibration period, intestinal motility was recorded over three 10-minute video recordings using Gastrointestinal Motility Monitoring system (GIMM; Med-Associates). Kymographs of intestinal diameter changes were generated using the video recordings (61). Kymographs were used to evaluate motor patterns in the colon.

CMMCs were defined as propagating contractions directed from the proximal to the distal end of the colon, which traveled more than 50% of the colon length (62–64). Frequency, velocity, and duration of CMMCs were analyzed using the GIMM processor plugin (ImageJ, NIH) (35, 65).

### Statistics.

Data analyses, except for quantitative comparisons of ENS composition, were performed using Prism 9 (GraphPad Software Inc.), and data are presented as mean ± SEM. Simple linear regression analysis was performed to determine the correlation between neurosphere number and length of intestinal resection. A 1-way ANOVA was performed with a post hoc Tukey’s test for multiple comparisons. For quantitative comparisons of ENS composition, Fisher’s exact test using R (66) was performed. *P* values were adjusted using Bonferroni’s correction. Survival analysis was performed using log-rank (Mantel-Cox) test. For all analyses, *P* values of less than 0.05 were regarded as significant.

### Study approval.

This study was conducted in accordance with the protocols reviewed and approved by the Institutional Animal Care and Use Committee at Massachusetts General Hospital (protocol 2009N000239). All methods were carried out in accordance with relevant guidelines and regulations. The reporting in the manuscript follows the recommendations in the ARRIVE guidelines.

### Data availability.

Values for all data points in graphs are reported in the Supporting Data Values file.

## Author contributions

RH, AJB, and AMG designed the research studies and edited and revised the manuscript. SB, WP, AAR, TO, and KO conducted experiments, analyzed data, and interpreted results of experiments. RS, LO, CH, and AL provided resources and technical support. RH, AAR, and TO drafted the manuscript. All authors approved the final version of manuscript.

## Supplementary Material

Supplemental data

Supplemental video 1

Supplemental video 2

Supplemental video 3

Supplemental video 4

Supporting data values

## Figures and Tables

**Table 1:**
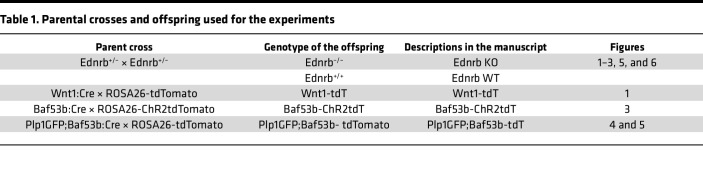
Parental crosses and offspring used for the experiments

## Version 1 07/23/2024

In-Press Preview

## Version 2 09/10/2024

Electronic publication
