# Supplementary material for: Enteric neural stem cell transplant restores gut motility in mice with Hirschsprung disease
Source: JCI Insight. 2024 Jul 23;9(17):e179755. doi: 10.1172/jci.insight.179755 (PMC11385093; doi:10.1172/jci.insight.179755)
Supplement: Supplemental data [file jciinsight-9-179755-s143.pdf]

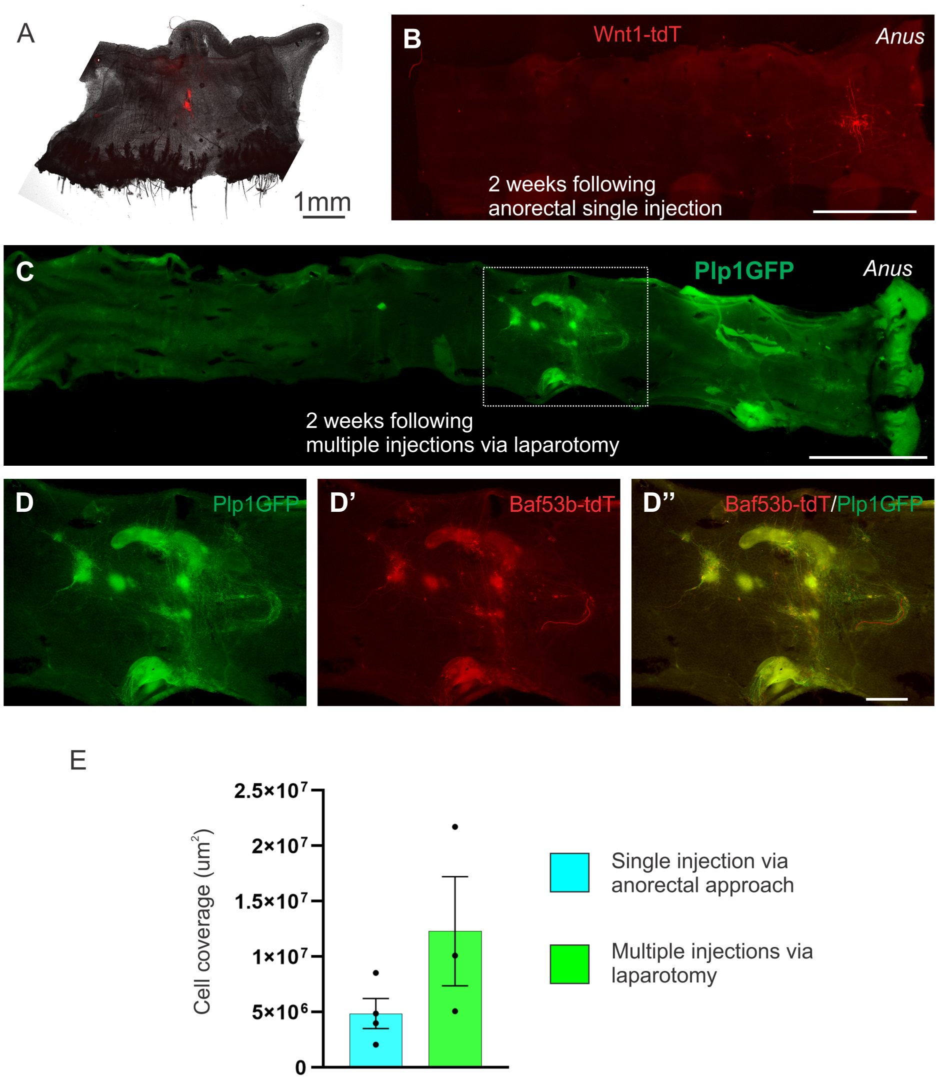

Supplementary Figure 1. Comparisons between single and multiple injections of ENSCs by two different approaches to Ednrb KO mice.
Transplanted cells were observed 2 weeks following single injection of ENSCs through an anorectal approach (A and B) or multiple injections via laparotomy (C). Dotted box in C was magnified in D-D'''. Area of cell coverage was measured (E).
Data shown as mean ± SEM. *$p < 0.05$ by Student's *t*-test.
Scale bars: 1 mm (B, D), 3 mm (A), and 5 mm (C).

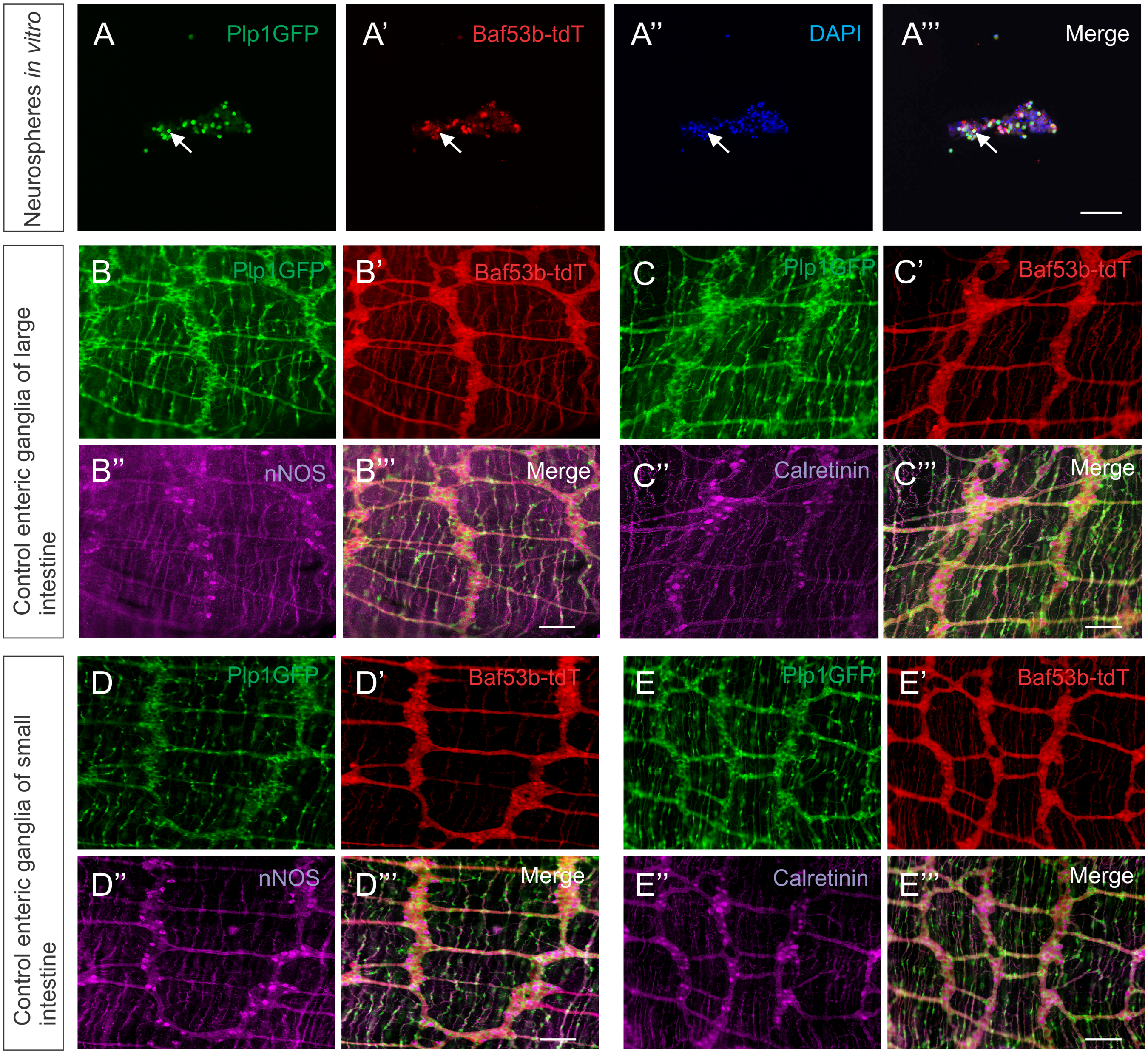

Supplementary Figure 2. Immunohistochemical characterization of ENSCs in culture and of enteric ganglia of wildtype mice.
Immunohistochemical evaluation of neurospheres isolated from the small intestine of Plp1GFP;Baf53b-tdT mice (A), and enteric ganglia of large (B-C) or small intestine (D-E) of Plp1GFP;Baf53b-tdT mice.
Scale bars: 100 μm (A-E).

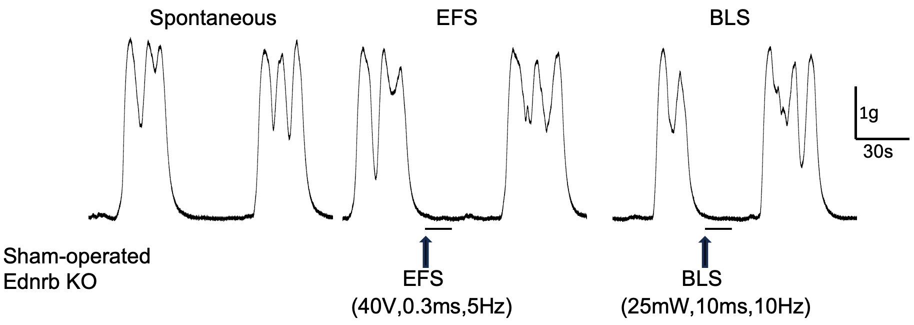

Supplementary Figure 3. Muscle contractility of sham operated Ednrb KO mice in response to electrical or optogenetic stimulation.

Representative traces of smooth muscle contractions during the “spontaneous”, after “EFS”, and “BLS”.

EFS, electrical field stimulation; BLS, blue light stimulation.

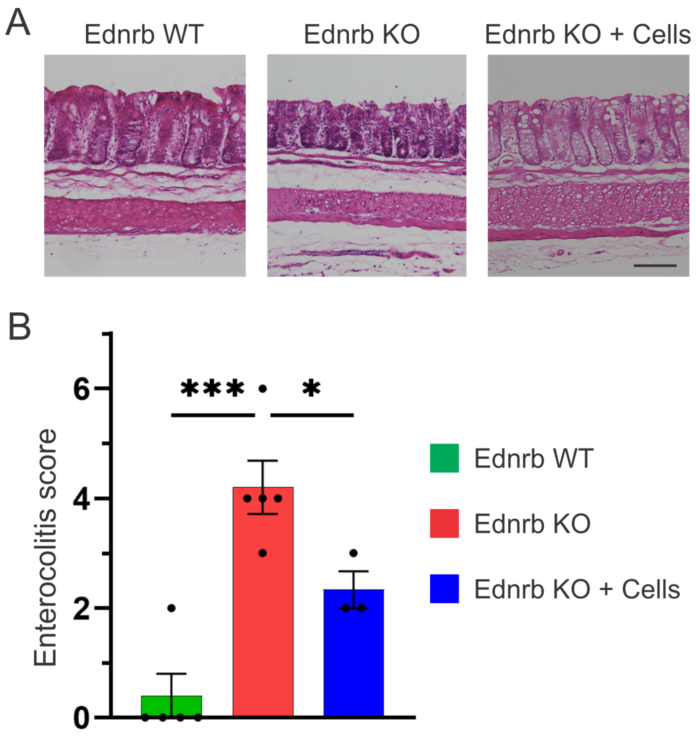

Supplementary Figure 4. ENSC transplantation reduces colonic inflammation and restores altered epithelial architectures in Ednrb KO mice.

(A) Histological examinations of distal colon of Ednrb WT, KO, or KO mice with ENSC therapy were performed and enterocolitis scores were evaluated (B).

All the values represent the mean of 2-4 animals for each group, repeated 2-3 times, and error bars represent the SEM. Statistical significance was determined by the one-way ANOVA with a post-hoc Tukey's test and *P* values ***$P < 0.001$, *$P < 0.05$ are statistically significant.
